# Characterization of fermented foods: bone health

**DOI:** 10.3389/fnut.2025.1648775

**Published:** 2025-08-29

**Authors:** Jyoti Prakash Tamang, Fojan Agahi, Birsen Yilmaz, İbrahim Ender Künili, Julie Mardon, Tuğçe Bulmus-Tuccar, Aleksandra Torbica, Daniela Nikolovska Nedelkoska, Mary-Liis Kütt, Jeadran Malagón-Rojas, Mayra Alejandra Parada, Baltasar Mayo, Juana Frias

**Affiliations:** ^1^Department of Microbiology, Sikkim University, Gangtok, Sikkim, India; ^2^Instituto de Ciencia y Tecnología de Alimentos y Nutrición (ICTAN-CSIC), Madrid, Spain; ^3^Department of Biological Sciences, Tata Institute of Fundamental Research, Hyderabad, India; ^4^Department of Fishing & Fish Processing Technology, The Faculty of Marine Sciences and Technology, Çanakke Onsekiz Mart University, Çanakkale, Türkiye; ^5^Université Clermont Auvergne, INRAE, Lempdes, France; ^6^Arla Innovation Center, Arla Foods, Aarhus, Denmark; ^7^Institute of Food Technology, University of Novi Sad, Novi Sad, Serbia; ^8^Faculty of Technology and Technical Sciences, University "St. Kliment Ohridski"-Bitola, Veles, North Macedonia; ^9^äio tech OÜ, Tallinn, Estonia; ^10^Observatorio Nacional de Salud, Instituto Nacional de Salud, Bogotá, Colombia; ^11^Grupo de Salud Ambiental y Laboral, Instituto Nacional de Salud, Bogotá, Colombia; ^12^Instituto de Productos Lácteos de Asturias (IPLA-CSIC), Oviedo, Spain; ^13^Investigación Sanitaria del Principado de Asturias (ISPA), Oviedo, Spain

**Keywords:** fermented foods, bone health, phytase, bio-peptide, vitamin K, gut-bone

## Abstract

Fermented foods are increasingly recognized for their potential benefits in supporting bone health, attributed to their rich content of bioactive compounds including vitamins K and B, polyphenols, peptides, and fermentation-modified phytates. This review examines how these components, enhanced in bioavailability through fermentation, may modulate bone metabolism via multiple mechanisms: improving mineral absorption, reducing inflammation, regulating oxidative stress, and influencing osteoblast and osteoclast activity. Special attention is given to the gut-bone axis, where fermented foods interact with gut microbiota to produce metabolites such as short-chain fatty acids and immunomodulatory compounds that may further support skeletal health. While preclinical and population-level studies show promising associations, clinical evidence remains limited and heterogeneous. Future research should focus on human trials, strain-specific effects, and long-term outcomes to fully establish the role of fermented foods in osteoporosis prevention and bone health maintenance.

## Introduction

Fermentation is an ancient method for food preservation and for producing new food items. Fermented foods worldwide are grouped according to the local ingredients available and the indigenous techniques employed to produce the edible products with desired sensory properties, which are named in accordance with local custom. These groups encompass fermented dairy, fermented grains, fermented meats, fermented fish, fermented legumes, fermented soybeans, fermented vegetables, fermented roots like cassava, and others (Table 1) (1).

**Table 1:** Native names of some traditional fermented foods of the world (1).

Fermented products	Continent-wise				
	Asia	Africa	Europe and Australia	North America	South America
Fermented dairy	Airag, Chhu, Chhurpi, Churkam, Dahi, Dadih, Kalari, Lassi, Mar, Misti dahi or Lal dahi, Mohi, Philu, Shrikhand, Somar, Sua Chua, Tarag	Amabere, Amaruranu, Amasi, Ergo, Fènè, Gariss, Kule Naoto, Leben, Lben, Mabisi, Mafi, Masai, Mursik, Mutandabota, Nunu, Omashikwa, Pendidam, Nyarmie, Sethemi, Suusac, Zabady	Hundreds of traditional European cheeses as a general term with various local names (Camembert, Cheddar, Brunost, Dubliner, Kefir, Koumiss, Manchego, Serra da Estrela, Skyr, Västerbottensost); Viili, Tarhana, yogurt	Cheese, yogurt	Cheese: Coalho, Corrientes, Minas, Pategrás, Reggianito Argentino Serrano; Yogurt
Fermented cereal	Ang-kak, Appam, Dosa, Idli, Jalabi, Khamak (Kao-mak), Lao-chao, Nan, Puto, Rabadi, Selroti, Tape Ketan	Busa, Ben-saalga, Enjera/Injera, Gowé, Hussuwa, Kenkey, Kunu-zaki, Kisra, koko, Mawè, Mbege, Ogi, Pito, Poto poto, Togwa, Uji	Sourdough, loaves	Sourdough, loaves,	Pozol, Sourdough
Fermented Meat	Arjia, Chartayshya, Kargyong, Khyopeh, Nham (Musom), Nem-chua, Pastirma, Sai-krok-prieo, Sa-um, Satchu, Suka ko masu, Tocin	Basterma, Basturma Gueddid, Khlii, Khlia, Msrana, Merguez, Naqaneq, Pastirma, Pastrami, Sujuk, Soudjouk	Hundreds of lesser-known and common traditional fermented sausages such as Alheira, Androlla, Chorizo, Morcilla, Pastirma, Peperoni, Salchichon/saucisson, Salsiccia, Soppressata, Sucuk; Hams,	Fermented sausages, ham, jerki	Sausages
Fermented Fish	Balao-Balao (Burong Hipon Tagbilao), Bordia, Belacan (Blacan), Bakasang, Burong Bangus, Budu, Gnuchi, Gulbi, Hentak, Hoi-Malaeng, Ika-Shiokara, Jeotkal, Karati, Kusaya, Lashim, Myulchijeot, Ngari, Narezushi, Nam Pla (Nampla-Dee, Nampla-Sod), Nuoc Mam, Patis, Pla-Paeng-Daeng, Pla-Som (Pla-Khao-Sug), Pu-Dong, Saeoo Jeot, Sheedal, Sidra, Suka ko Maacha, Sukuti, Shottsuru, Sikhae, Surströmming, Tungtap	Feseekh, Momone	Surströmming, rakfisk, hákarl	Smoked fish	Smoked fish
Fermented Legume	Bhallae, Dhokla, Maseura, Oncom Hitam (Black Oncom), Oncom Merah (Orange Oncom), Papad, Wari	Bikalga, Dawadawa, Iru, Kawal, Kinda, Ogiri, Ogili, Okpehe, Soumbala, Ugba	Unconsumed	Unconsumed	Unconsumed
Fermented Soybean	Axone/Aakhoni, Bekang, Chongkukjang, Doenjang, Douchi, Furu, Grep Chhurpi, Gochujang, Hawaijar, Kinema, Kanjang, Kecap, Ketjap, Natto, Meitauza, Meju, Miso, Pe poke, Peruyaan, Peron Naming, Pheha Shoyu, Sieng, Sufu, Tauco, Thua Nao, Tempe, Tungrymbai, Yandou	Not consumed	Unconsumed	Unconsumed	Unconsumed
Fermented vegetable	Burong mustala, Dha muoi, Ekung, Eup, Fu-tsai, Gundruk, Goyang, Hom-dong, Hiring, khalpi, Jiang-gua, Jiang-sun, Kimchi, Naw-mai-dong, Mesu, Oiji, Pak-gard-dong, Pak-sian-dong, Pao cai, Soidon, Soibum, Sayur asin, Sinki, Sunki, Suan-cai, Suan-tsai Takuanzuke, Tuaithur	Fermented olives	Sauerkraut; Sapal (fermented *Colocasia esculenta* and coconut) in Papua New Guinea	Sauerkraut	Fermented olives
Fermented roots (cassava)	Tape	Chikwangue, Cingwada, Fufu, Gari, Lafun, Konkonte	Unconsumed	Unconsumed	Beiju, *Calugi*, Cauim, *Caxiri*, Puba, *Tarubá*, Tucupi, *Yakupa*, Y *Parakari*

The authors express regret for any errors in the spelling or phonetics of local names for traditional fermented foods, should they be incorrect.

In regions with widespread pastoral farming, such as the Middle East, Europe, and the Indian subcontinent, abundant milk from cows, sheep, and goats led to the development of fermented dairy products like fermented milk and cheese (2). In contrast, East Asian countries like China, Japan, and Korea, where animal husbandry was less prominent, developed fermented fish and soy-based foods instead (3, 4). In Africa, fermentation traditions rely on local grains (millet, sorghum, maize, wheat) and roots like cassava, alongside naturally fermented dairy from cows, buffalo, and camels (5, 6). Similarly, in Europe, the Americas, and Australia, livestock farming has supported the production of fermented dairy (7) and meat products (8), which are an integral part of local diets.

Traditionally, foods that undergo natural or spontaneous fermentation are produced from either plant or animal origins that do not require starter cultures. Numerous fermented foods are still produced via natural or spontaneous fermentation (9) or by employing back-slopping methods (10, 11). Many traditional fermented foods continue to be made at home, on a small scale, and with traditional practices. Nevertheless, the 20th century witnessed a substantial rise in the availability of starter cultures, which are now commonly used in commercial fermentation processes for products such as wine, dairy, and meat, particularly in Western nations. The application of starter cultures, utilizing the functional strains of food-grade bacteria, yeasts, or molds, represents a major transformation in the fermented foods and beverages market (12).

Historically, the main role of fermented foods was for preservation, but their extra advantages became noticeable as time passed. Prior to the establishment of nutrition science, fermented foods were made to ensure a consistent supply of vitamins, minerals, calories, and other vital nutrients required for survival. Progress in food microbiology and nutrition has indicated that specific beneficial microorganisms are essential in converting raw materials into fermented food products. These microorganisms aid in producing bioactive substances, vitamins, immune system enhancers, short-chain fatty acids (SCFAs), and secondary metabolites, which all contribute to consumer health and well-being (13–15). Genomic techniques supported by multi-omics approaches are increasingly being employed to investigate diverse microbial communities and their bio-functionalities present in fermented foods (16, 17), as well as to detect unidentified biomarkers or genetic signatures for various health and therapeutic purposes (18).

Fermented foods present numerous health benefits, which include improved digestion and regulation of the gut microbiome (19–21), prevention of type 2 diabetes and metabolic syndrome (22, 23), decreased risk of cardiovascular diseases (24), relief from lactose intolerance (25), reduction of inflammation (26), combating obesity (27–29), aiding bone healing (30, 31), exhibiting anti-cancer properties (32, 33), and preventing neurodegenerative diseases (34). Fermented foods are also positioned as key dietary ingredients to promote bone health. Since bone-related diseases represent a wide burden worldwide, it is of great importance to investigate the ability of fermented food to preserve bone health. Indeed, for instance, osteoporosis has a significant impact on individuals’ quality of life and morbidity worldwide, particularly among the elderly and postmenopausal women. Given the increase of the number of bone fractures during the last decades as well as the associated burden to individuals, families, societies, and health-care systems (35, 36), the prevention of bone diseases is a public health priority. Thus, a thorough investigation of the links between fermented foods and bone health is essential. Among these emerging benefits, bone health has gained attention as a novel and promising target for dietary modulation through fermented foods. This interest arises from several factors. First, osteoporosis and low bone mineral density (BMD) are major global public health concerns, particularly affecting aging populations and postmenopausal women (36–38). Second, diet plays a pivotal role in modulating bone metabolism, and the gut–bone axis has emerged as a key mediator linking dietary habits to skeletal outcomes (39). Recent evidence suggests that fermented foods through their unique matrix of nutrients, microbial metabolites, and live microorganisms may influence bone health beyond their basic nutritional content (40–42). This influence is hypothesized to occur through various mechanisms including modulation of gut microbiota, reduction of systemic inflammation, and enhancement of mineral bioavailability (43, 44).

Bone is a dense and structured tissue made up of cells surrounded by a plentiful hard intercellular substance that consists of collagen fibers and calcium phosphate (45). The growth, preservation, and renewal of bone tissues within the human body involve a series of co5rdinated mechanisms that result in tissue development, maintenance, and healing after injury (46). Bone metabolism necessitates essential dietary micronutrients like calcium (47), phosphorus (48), magnesium (49), and vitamin D (50). Minerals are taken up in the upper section of the gastrointestinal tract in an ionized form, which is the typical state under the stomach’s low pH. The uptake of calcium is significantly enhanced by vitamin D, making this vitamin essential for its availability in the body (51). Research has also linked vitamins B complex and K as important factors in maintaining bone health (52). The way B vitamins influence bone physiology seems to be related to their impact on homocysteine metabolism (53), while the action of vitamin K appears to occur through the steroid xenobiotic receptor and/or through the *γ*-carboxylation of osteocalcin (OC), which is the most prevalent vitamin K-dependent protein specific to bone (54).

In recent years, fermented foods have attracted attention as possible dietary interventions for bone health because of their rich content of bioactive compounds (40–42, 55, 56). Fermentation is a microbial biochemical process that produces numerous metabolites (organic acids, peptides, vitamins, etc.) from the macromolecules of raw materials, some of which may influence bone health (57–59). Certain fermented foods are particularly rich in group B vitamins (riboflavin and folates) and vitamin K (menaquinones) (60, 61), as they are predominantly produced by fermentative microorganisms, especially those belonging to lactic acid bacteria (LAB). In plant-based foods, the absorption of minerals is negatively impacted by the presence of anti-nutritional factors, such as oxalates and phytates (62); these are diminished during fermentation due to the action of microbial enzymes, simultaneously aiding in the release (and availability) of phosphorus (63). Additionally, the low pH of most fermented foods may delay the neutralization of the chyme, thereby prolonging ionization and enhancing absorption (64). Hence, the intake of fermented foods could assist the body in absorbing a greater amount of minerals and improving the intake of vitamins that promote bone health. Preclinical and population studies have suggested potential associations between fermented food consumption and improved bone density or reduced fracture risk (65–67), however, the multiple underlying mechanisms ranging from enhanced nutrient bioavailability and microbiota modulation to anti-inflammatory effects, have not been comprehensively investigated within a single framework.

Phytic acid, vitamins B complex and K, polyphenols, and bioactive peptides are essential biological compounds affecting bone health in humans (68–71). These compounds whether naturally present, enriched, or modified through fermentation, are central to understanding how fermented foods may influence bone metabolism. Recently, it has been reported that fermented foods may enhance bone health via the gut microbiota by stimulating the osteoimmune system, producing SCFAs, and facilitating the absorption of nutrients such as calcium (72).

While most of these compounds are generated or enriched during fermentation, in some cases like phytic acid, fermentation contributes by reducing their levels, thereby indirectly supporting mineral bioavailability and bone health. Despite this reduction, phytic acid itself has been reported to exert context-dependent effects on bone metabolism, and is therefore discussed as a relevant compound within the scope of this review.

**Figure 1:**
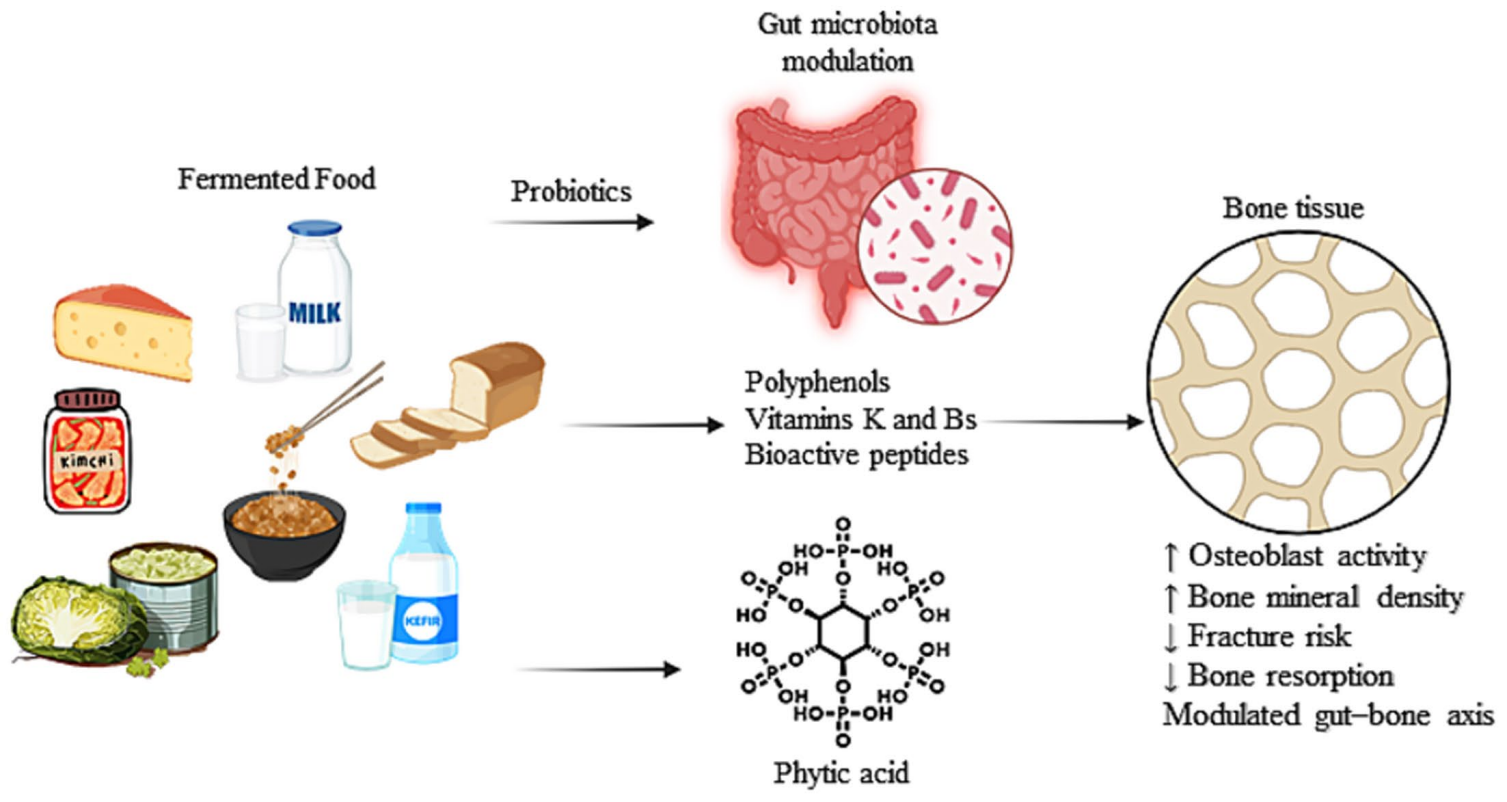
Schematic representation of the effects of fermented food-derived bioactive compounds and microbial modulation on bone metabolism.

However, despite increasing interest, the body of evidence remains fragmented across food types, health claims, and mechanisms. A comprehensive review is thus warranted to systematically explore and consolidate current knowledge on the potential of fermented foods in maintaining or improving bone health, their mechanisms of action, and their relevance in dietary strategies for osteoporosis prevention and bone metabolism modulation. Hence, we attempt to review the various beneficial effects of consuming fermented foods on bone health and the molecular underpinnings of the various mechanisms by which these benefits are conferred. The proposed mechanisms by which fermented foods and their bioactive compounds may support bone health, primarily through modulation of gut microbiota and enhancement of nutrient bioavailability, are illustrated in Figure 1.

## Phytic acid and bone health

Phytic acid (C₆H₁₈O₂₄P₆), also known as inositol hexaphosphate, inositol hexakisphosphate, or inositol polyphosphate, is a naturally occurring compound consisting of six dihydrogen phosphate groups linked to inositol (73). At physiological pH levels commonly found in plants, phytic acid exists predominantly as phytate anions, specifically myo-inositol-1,2,3,4,5,6-hexakisphosphates (74). These compounds serve as major phosphorus storage molecules in plant seeds, accounting for approximately 10% of total plant weight and over 60% of total phosphorus content, particularly in cereals, legumes, oilseeds, and nuts (75–77). The structural characteristics of phytates—featuring negatively charged phosphate groups arranged around inositol rings—enable them to form strong chelation complexes with divalent cations, including zinc, iron, calcium, magnesium, manganese, and copper (78, 79). This mineral-binding capacity has traditionally classified phytates as antinutrients, as consumption of raw phytate-rich plant materials can significantly reduce the bioavailability and absorption of essential minerals (80).

However, recent research has fundamentally challenged the traditional view of phytic acid as solely an antinutrient, revealing complex beneficial effects on bone metabolism. Studies have demonstrated that phytic acid contributes to bone development and helps mitigate age-related deterioration of bone marrow mesenchymal stem cells (BMSCs), particularly under hyperglycemic conditions. In diabetic environments, phytic acid enhances osteogenic differentiation and suppresses oxidative stress-induced cellular senescence, primarily through activation of the extracellular signal-regulated kinase signaling pathway (70).

The molecular mechanisms underlying these beneficial effects have been further elucidated through investigations of phytic acid’s role in bone regeneration under diabetic conditions. Phytic acid supplementation restores the osteogenic capacity of BMSCs by modulating the expression of circular RNA (circRNA) circEIF4B (a non-coding RNA that regulates gene expression by acting as a microRNA sponge). This regulatory mechanism promotes osteogenesis by sequestering miR-186-5p and upregulating forkhead box protein O1, while simultaneously stabilizing integrin subunit alpha 5 (ITGA5) mRNA through inhibition of insulin-like growth factor 2 mRNA-binding protein 3 (IGF2BP3) degradation. Notably, the inhibition of circEIF4B impairs the bone-regenerative effects of phytic acid *in vivo*, confirming the functional importance of this molecular axis in diabetes-related bone disorders (81). Clinical evidence supports these mechanistic findings. In a Mediterranean cohort of postmenopausal women, dietary phytate intake showed positive associations with bone mineral density at multiple skeletal sites, including the femoral neck, total femur, and lumbar spine. This protective effect was most pronounced in women under 66 years of age without type 2 diabetes, with the proposed mechanism involving phytate adsorption onto hydroxyapatite surfaces, thereby inhibiting bone resorption (82).

Additional studies have demonstrated that phytate functions as both a crystallization and dissolution inhibitor by adsorbing onto hydroxyapatite crystal surfaces. *In vitro* experiments revealed that the anti-resorptive effects of phytate were comparable to alendronate and superior to etidronate. Cross-sectional analyses further showed that phytate intake above 307 mg/day was associated with normal bone mineral density levels in the lumbar spine of postmenopausal women (83).

Clinical interventions have provided additional support for phytate’s bone-protective properties. Oral supplementation with calcium–magnesium phytate (InsP6) significantly reduced urinary calcium excretion and serum *β*-CrossLaps levels—a marker of bone resorption—in hypercalciuric patients with osteopenia or osteoporosis, suggesting that phytate may help prevent bone demineralization in individuals with excessive bone turnover (84).

Long-term fracture risk assessment has provided compelling evidence for phytate’s protective role. A clinical study evaluating the relationship between urinary phytate concentrations and 10-year fracture risk in recently postmenopausal women demonstrated that those with higher urinary phytate levels (≥1.0 mg/L) had significantly lower FRAX-predicted risks of major osteoporotic and hip fractures compared to women with low phytate levels (≤0.50 mg/L). This protective effect was particularly pronounced in women with established osteoporosis risk factors, such as tobacco or alcohol use, suggesting that higher intake of phytate-rich foods may reduce long-term fracture risk in at-risk populations (85).

While accumulating evidence supports the beneficial effects of phytic acid on bone health, its dual nature requires careful consideration. Recent comprehensive reviews have summarized both the beneficial and potentially adverse effects of phytic acid (IP6) on bone metabolism (86). Although traditionally considered an antinutrient due to its mineral-chelating capacity, IP6 has been shown to bind to hydroxyapatite crystal growth sites, inhibiting pathological calcification. However, this same mechanism raises legitimate concerns about possible interference with physiological bone mineralization processes. Current evidence predominantly supports a protective role of IP6 in bone health through modulation of both extracellular crystallization and intracellular signaling pathways, but the balance between beneficial and potentially harmful effects may depend on dosage, timing, and individual physiological conditions (86).

Understanding this complexity becomes particularly important when considering food processing methods that can modify phytate levels. The breakdown of phytate is mediated by phytase enzymes, which are absent in humans and non-ruminant animals (87). These enzymes occur naturally in plants and microorganisms and exhibit optimal activity at pH levels near 5, though they remain active across pH ranges of 2 to 8 (88). The effectiveness of phytases in degrading phytate can be enhanced through various food processing methods, including cooking, soaking (89), germination (90), and fermentation (91, 92).

Among these methods, fermentation offers particular advantages for promoting bone health by simultaneously reducing pH and enhancing phytase activity (93, 94). The acidic environment created during fermentation not only activates phytases more effectively but also improves calcium absorption—a critical factor for maintaining skeletal integrity (95). For example, sourdough bread made from specific vegetable flours exhibits lower pH compared to original ingredients (96), resulting in up to 45% reduction in phytate content after baking (97). Similarly, vegetable fermentation processes, such as those used to produce kimchi or sauerkraut, reduce pH from approximately 3.9 to 4.4 (98).

The reduced phytate levels in fermented foods compared to raw components (73, 79) can significantly improve mineral bioavailability (Table 2). Studies have demonstrated increased calcium bioavailability in yogurt made from soy milk, with potential benefits for bone health (99). Animal studies have provided additional support, showing that ovariectomized rats fed fermented soybeans with *Bacillus subtilis* exhibited enhanced bone mineral density after 12 weeks (100). These findings suggest that dietary intake of appropriately fermented foods can help maintain bone health by optimizing the balance between phytate’s beneficial effects and mineral availability.

**Table 2:** Key fermented foods, their bioactive compounds, and mechanisms influencing bone health.

Fermented food category	Fermented food	Key minerals	Phytate factor	Bioactive compounds	Mechanisms of action on bone health	References
Fermented dairy	*Yogurt*	Calcium, Magnesium, Phosphorus	Phytate: Not present	Vitamins B2, B12. BAPs (from casein digestion), exopolysaccharides, conjugated linoleic acid (if full-fat), SCFAs (via gut microbiota).	↑Mineral solubility; ↑Vit B2, B12 (LAB); peptides ↑Ca absorption & ↓resorption; EPS ↑mineral bioavailability; CLA may ↑bone formation; SCFAs modulate gut–bone axis.	(220–223)
	*Kefir*	Calcium, Magnesium, Phosphorus	Phytate: Not present	Vitamins B1, B2, B12, Vitamin K2 (MK-7, MK-9). BAPs, exopolysaccharides (especially kefiran), conjugated linoleic acid (if full-fat), SCFAs (via gut microbiota), organic acids, polyamines.	Org. acids & LAB ↑Vit B; K2 activates osteocalcin; peptides ↑Ca absorption & ↓resorption; kefiran & polyamines ↑gut integrity; SCFAs modulate gut–bone axis.	(61, 224, 225)
Fermented vegetables	*Kimchi*	Calcium, Iron, Phosphorus	Phytate: 0.2% → <0.1%	Vitamin C, B vitamins, Vitamin K2 BAPs, SCFAs (via gut microbiota), polyphenols, isothiocyanates, organosulfur compounds, conjugated linoleic acid (trace), GABA, lactic acid.	↓Phytates, ↑mineral bioavailability; ↑Vit C & K2 (LAB); peptides & GABA ↑osteoblasts; polyphenols & sulfur comps ↓oxidative stress; SCFAs & lactic acid ↑Ca absorption.	(226, 227)
	*Sauerkraut*	Potassium, Calcium, Magnesium	Phytate: 0.3% → <0.1%	Vitamin C, vitamin K2. BAPs, SCFAs (via gut microbiota), polyphenols, glucosinolates and their breakdown products (e.g., isothiocyanates), lactic acid.	Phytate reduction ↑mineral bioavailability; Vit C preserved, K2 ↑ (bacteria); peptides aid Ca uptake; glucosinolate derivatives & polyphenols ↓inflammation; lactic acid & SCFAs boost mineral solubility and gut health.	(228, 229)
Fermented grains	*Sourdough*	Iron, Zinc, Magnesium	Phytate: 1.2% → 0.5%	Vitamins B1, B6, B9. Organic acids (lactic, acetic), BAPs (from gluten and other proteins), phenolic compounds, B vitamins (produced by lactic acid bacteria), phytase enzyme.	Phytate ↓ (up to 45%) boosts mineral absorption; B vitamins ↑ support energy & bone health; phytase breaks down phytates; organic acids enhance mineral solubility; BAPs aid calcium uptake; phenolics provide antioxidant support.	(230–232)
Fermented soya	*Miso*	Sodium, Calcium, Potassium	Phytate: 1.5% → 0.5%	Vitamins B2, B12, Vitamin K2 (MK-7, MK-9). Isoflavones (e.g., genistein, daidzein), BAPs, polyamines, SCFAs (via gut microbiota), B vitamins, saponins.	Long fermentation ↑Ca & K bioavailability; synthesizes Vitamins B & K2, aiding osteocalcin activation. Isoflavones mimic estrogen, modulating bone metabolism; BAPs ↑Ca absorption; polyamines & saponins support gut & mineral uptake; SCFAs ↓ inflammation, promote bone remodeling	(233, 234)
	*Tempe*	Iron, Zinc, Calcium, Magnesium	Phytate: 2.1% → 0.6%	Vitamins B2, B12, Vitamin K2 (MK-4). Isoflavones (genistein, daidzein), BAPs, polyphenols, B vitamins, SCFAs (via gut microbiota), saponins.	70% phytate ↓ boosts Fe/Zn bioavailability; K2 (mainly MK-4) supports bone formation. Isoflavones modulate bone via estrogen receptors; BAPs aid Ca absorption; polyphenols/saponins ↓oxidative stress; SCFAs ↑ gut health and mineral uptake.	(235, 236)
	*Natto*	Calcium, Iron	Phytate: 1.8% → 0.5%	Vitamin K2 (MK-7), nattokinase, isoflavones, BAPs, polyamines, B vitamins	Phytate ↓ boosts Ca/Fe absorption; high MK-7 ↑osteocalcin carboxylation, strengthening bones and reducing fracture risk. Isoflavones regulate bone turnover via estrogen receptors; nattokinase supports bone perfusion; BAPs and polyamines aid Ca uptake and metabolism.	(233, 234)
Fermented fish	*Plaa-som*	Calcium, Phosphorus	Phytate: Not present	Vitamin B12, D, K2 BAPs, omega-3 fatty acids, B vitamins, SCFAs (via gut microbiota)	Preserves minerals; ↑Ca & P bioavailability. Vitamins B12, D, K2 support bone health. BAPs aid mineral absorption and inhibit bone resorption. Omega-3 s ↓inflammation and promote remodeling. B vitamins regulate homocysteine and maintain bone matrix. SCFAs improve gut health and nutrient uptake	(237–239)
	Fish sauce	Calcium, Sodium	Phytate: Not present	Vitamin B12, vitamin K2. BAPs, amino acids (e.g., glutamic acid), trace minerals (calcium, phosphorus).	Mineral extraction and B12 synthesis ↑ nutrient absorption; high sodium content noted; trace K2 may aid bone health. BAPs support Ca absorption and ↓ resorption; amino acids aid protein metabolism and bone matrix; trace minerals support mineralization	(239, 240)

Short-chain fatty acids, SCFAs; Bioactive peptides, BAPs; γ-aminobutyric acid, GABA; lactic acid bacteria, LAB; conjugated linoleic acid, CLA.

Despite accumulating evidence on the bone-protective effects of phytate, its dual role as both a mineral chelator and functional bioactive compound presents important challenges. While fermentation has emerged as a promising strategy to reduce phytate content and enhance mineral bioavailability, the precise balance between retaining phytate’s benefits and mitigating its antinutritional effects remains unclear. The long-term impact of fermented phytate-rich foods on bone health also requires further investigation. Most existing studies are short-term or observational, limiting conclusions on causality and durability of effects. Additionally, individual differences in phytate metabolism, fermentation efficacy, and dietary patterns further complicate outcome consistency. Interactions between fermented foods and other nutrients, as well as the potential role of gut microbiota in modulating phytate activity post-ingestion, are underexplored areas warranting deeper investigation.

Given the phytic acid dual role as both a mineral chelator and a functional bioactive, it offers a compelling starting point to illustrate how fermentation transforms food components in ways that directly influence bone health. Its modulation during fermentation highlights one of the most fundamental biochemical mechanisms, enhanced mineral bioavailability that underpins the relevance of fermented foods in skeletal support. Building on this foundation, we next explore the role of other key bioactive compounds influenced by fermentation, including vitamins K and B complex, polyphenols, and bioactive peptides. Each contributes to bone health through distinct but complementary pathways, ranging from nutrient signaling and antioxidant activity to hormonal modulation and cellular communication. Finally, these effects converge through the gut-bone axis, where the gut microbiota plays a central role in mediating the systemic impact of fermented foods on bone metabolism.

## Vitamins K and B complex for bone health

Vitamin K is essential for maintaining bone health (101), which occurs in two varieties: vitamin K1 (phylloquinone) and vitamin K2 (menaquinones) (102). Vitamin K1 can be found in green leafy vegetables and certain plant oils (103), and vitamin K2 is synthetized by bacteria through fermentation or from animal-derived foods (104), which is critical for bone health (Table 2). For example, the level of vitamin K in *natto*, the Japanese fermented soybean food, with *Bacillus subtilis*, rises from 0.5 μg/g in soybeans to 10 μg/g in the finished product fermented with *Bacillus subtilis* (19). Vitamin K2 plays a crucial role in activating proteins referred to as Gla proteins, which encompass OC, one of the 17 Gla proteins found in humans. The activated variant of OC is significant for the development and preservation of bones (105). There are various forms of vitamin K2; for instance, the short-chain type, MK-4, is synthesized from vitamin K1 in the body and can be found in animal products. Longer-chain variants such as MK-7, MK-8, and MK-9 are synthetized by bacteria (106, 107) and are present in fermented milk foods like cheese (108, 109), as well as in fermented soybean products such as *natto* from Japan (110) and *kinema*, native fermented food from the Himalayas (111).

Many studies have explored the relationship between vitamin K intake and bone health. Some research shows inconsistent findings about how vitamin K supplements affect bone health markers (112, 113). Recent research, however, showed a clear link between low vitamin K intake and knee cartilage damage in people with severe knee pain and osteoarthritis, especially among women (114). Additionally, a higher dietary intake of vitamin K was linked to better bone quality and greater BMD (115). Recent research has shown that vitamin K2 supplements can help postmenopausal status by reducing bone loss and enhancing bone quality in Japanese women (116–118).

Numerous studies have investigated the relationship between vitamin K2 sourced from *natto* and bone health. The effects of vitamin K1 supplements containing MK-7, which is present in *natto* extract, demonstrated that MK-7 aids in the improved carboxylation of OC (110). Habitual intake of natto is linked to a reduced risk of osteoporotic fractures in Japanese postmenopausal women, independently of bone mineral density (119). Additionally, natto consumption is indirectly associated with lower rates of tooth loss—possibly via improved systemic bone density (120). Collectively, current evidence supports recommending natto to help prevent bone loss in both premenopausal and postmenopausal Japanese women (121, 122). Lundberg et al. (109) examined the influence of Jarlsberg and Camembert cheeses on bone health. They discovered that Jarlsberg cheese, which contains long-chain vitamin K2, enhances total serum OC levels, increases carboxylated OC, and improves the OC ratio, thus fostering bone growth. Within every 100 g of Jarlsberg cheese, there exists 3.0 μg of vitamin K1, 5.2 μg of MK-4, and 1.5 μg of MK (109).

Vitamin K is well-recognized for its role in maintaining bone density as individuals age; however, emerging research also highlights the importance of B vitamins in relation to osteoporosis and fracture risk (123). For instance, one study found that higher intake of vitamin B2 is significantly associated with a reduced risk of femoral osteoporosis and bone loss (124). In a large cohort of US women over the age of 50, elevated levels of homocysteine and methylmalonic acid, biomarkers of poor B-vitamin status, were linked to lower bone mineral density and an increased risk of lumbar spine osteoporosis, despite the widespread fortification of foods with B vitamins (125). These findings emphasize the role of adequate B-vitamin status in supporting bone health and reducing fracture risk. Additionally, a study involving 63,257 adults aged 45 to 74 years reported that higher pyridoxine (vitamin B6) intake was associated with a lower risk of osteoporosis. While several investigations have explored the potential benefits of B-complex vitamins on low bone mineral density, some studies suggest that these vitamins may not directly improve BMD outcomes (125, 126). A positive correlation exists between water-soluble vitamins and BMD, primarily associated with vitamins B12, and C. Conversely, a negative association was observed with fat-soluble vitamins, especially vitamins E and A (126).

Despite the growing body of literature on the role of vitamins K and B in bone health, other researchers have not found an association of unspecified vitamin K intake and bone fracture risk, e.g., (127) did not find any association between K1 or K2 intake and vertebral fractures in the Hordaland Health Study of 2,994 Chinese community-dwelling men and women aged 65 years and over in Hong Kong. Moreover, results from other studies showed an insignificant effect of vitamin K on lumbar spine and femoral neck BMD (128).

while B-vitamin deficiencies, especially B6, B12, and folate, are linked to elevated homocysteine levels and reduced BMD, causal evidence from intervention trials remains inconclusive. Results from several *in vitro* and *in vivo* studies were generally consistent and showed that, although B vitamin deficiency generated significantly higher serum level of homocysteine, there was no significant effect on bone strength and bone area, mineral matrix, callus stiffness, size or tissue composition or bone turnover (129). Similarly, In the Singapore Chinese Health Study, dietary intake of B1 and B3 was not associated with risk of hip fracture in either men or women (130).

The results from observational studies are inconsistent in the associations between K and B vitamins and bone outcomes, however, majority of the randomized clinical trials have not shown protective effects of these vitamins in bone turnover or fracture risk reduction. Hence, while observational and mechanistic studies suggest a potential role for vitamins K and B in bone health, current evidence from randomized clinical trials remains insufficient to support strong clinical recommendations, highlighting the need for well-designed, long-term intervention studies to clarify their therapeutic relevance.

## Polyphenols for bone health

In fermented plant-based foods, such as whole-grains, vegetables, and fruits, polyphenols are the main bioactive compounds (131). Generally categorized as flavonoids or non-flavonoids, polyphenols represent a group of secondary metabolites present in plants that exhibit various biological activities (132). According to their structural features, flavonoids can be divided into flavanols, flavones, flavanones, chalcones, and isoflavones (133). Non-flavonoids consist of lignans, astragalus, tannins, and phenolic acids, among others (131).

Numerous studies have reported the beneficial influence of polyphenols on the prevention and treatment of bone health. Overall, the intake of polyphenols may aid in preventing bone loss and decrease fracture risk (134), providing a potential strategy for preventing and managing osteoporosis by impacting bone metabolism, decreasing bone resorption, sustaining bone density, and diminishing osteoclast differentiation (3), while also fostering bone formation and hindering bone resorption (135).

Li et al. provided a theoretical framework for the mechanisms of polyphenol compounds related to bone formation and absorption (136). Polyphenols provide advantageous effects on osteoporotic bone defects by regulating oxidative stress, reducing inflammation, promoting osteogenesis, inhibiting osteoclast differentiation, and inducing osteoclast apoptosis (137).

The fermentation of food is crucial for increasing the beneficial impacts of polyphenols in food (Table 2), as polyphenols that aren’t fermented cannot be absorbed by the intestines, significantly limiting their bioavailability (138). Fermentation enhances the bioaccesibility and bioavailability of polyphenols by transforming intricate polyphenols in food into more straightforward, absorbable forms via beneficial microorganisms and enzymes related to polyphenols (131). These small-molecule free phenols exhibited greater biological activities and bioavailability compared to the macromolecular-bound phenols (131).

In accordance with the aforementioned the results indicate that the total polyphenols amount increases by 30.3% during spontaneous fermentation in the production process of *cheonggukjang,* a fermented soybean food of Korea (139), and in vinegar made from black wolfberry by 42.9% (140). During the fermentation of *cheonggukjang*, the total content of flavonols and phenolic acids rises due to the hydrolysis process that converts isoflavone glycosides into aglycones (141). Following a short fermentation period, the levels of aglycone-type isoflavones (daidzein, glycitein, and genistein) increased, while the levels of glycoside-type, malonyl-, and acetyl-type isoflavones decreased (142). This evidence verifies that *cheonggukjang* serves as a significant source of isoflavone aglycones like daidzein and genistein, even with the brief fermentation duration of soybeans (141). The effectiveness of *cheonggukjang* in preventing bone mass loss due to osteoporosis was demonstrated by observing reduced bone length and loss in animals fed a diet containing 10% *cheonggukjang* over a period of 15 weeks (143). According to (144), total isoflavone glycosides decreased and total isoflavone aglycones increased in 70% methanol extracts of three types of standardized *chungkookjang* inoculated with 1% (v/w) *B. subtilis, B. licheniformis*, or *B. amyloliquefaciens* in comparison with unfermented cooked soybeans.

Also, according to the prior research, the bioavailability and bioactivity of isoflavonoids are increased with fermentation, since *in vivo* studies have shown that isoflavone aglycones (daidzein and genistein) were absorbed more effectively in the human intestine by consuming *tempe,* Indonesian fermented soybean food, as opposed to a soybean-pieces diet (145, 146). Recently, soy pulp, which is a by-product of black soybean food processing, has been utilized as animal feed or discarded as waste. Among fermented black soybean products, fermented black soybean pulp supports bone metabolism and osteoporosis prevention (147). The research conducted by (147) indicated that genistein levels in black soybean pulp after 12 and 24 h of fermentation with *L. acidophilus* were 6.8 and 7.2 times higher, respectively, in comparison to controls. One group of ovariectomized rats was treated with black soybean pulp, while a second group of ovariectomized rats was treated with fermented black soybean pulp, indicating that the fermentation of black soy pulp is effective in preventing osteoporosis in ovariectomized rats (147). Finally, numerous studies have shown that (−)-epigallocatechin-3-gallate (EGCG), (−)-epicatechin gallate (ECG), and epigallocatechin (EGC) present in fermented tea are the main compounds that influence bone mass and prevent bone loss (148).

Recent intervention studies investigating the effects of polyphenols, consumed via food or given as isolated compounds, showed inconsistent results regarding bone health (Table 2). While some studies suggest that dietary intervention with polyphenol rich foods may be useful to prevent the incidence and progression of osteoporosis (149), on the other hand, evidence from human intervention studies does not allow a clear conclusion on the effects of dietary polyphenols on bone mineral density and bone turnover markers (150). Consequently, conclusive determinations cannot be reached. The current evidence of polyphenols’ impact on bone health prevention and treatment is based on numerous review articles that address findings from previous decades. The understanding of polyphenols’ effects related to bone health is limited due to the lack of evidence in recent research.

Alongside polyphenols, fermentation also generates bioactive peptides with demonstrated potential to influence bone remodeling. These peptides derived mainly from dairy and soy protein hydrolysis, function through additional pathways such as calcium binding, hormone modulation, and direct stimulation of osteoblast activity, complementing the effects of both polyphenols and vitamins.

## Bioactive peptides for bone health

Bioactive peptides (BAPs) are initially encoded within their parent proteins and are released through enzymatic hydrolysis during fermentation and/or digestion (151). BAPs possess various beneficial effects on bone health (71, 152). Fermented foods are recognized for containing BAPs that are derived from the fermentation process by the actions of bacteria such as LAB, along with some *Bacillus* species (153–155). Nonetheless, there is minimal research regarding how these BAPs influence bone health.

In dairy products, the primary proteins acting as sources for generating BAPs are caseins and whey proteins (156). These peptides typically emerged from the proteolytic activity of LAB (157–160). Employing an untargeted peptidomics approach, researchers discovered BAPs exhibiting antioxidant, antihypertensive, and antidiabetic properties after fermenting whey protein concentrate with strains of *Lactobacillus helveticus* and *Streptococcus thermophilus* (161). Specific peptide sequences such as Ile-Pro-Pro and Val-Pro-Pro, which are liberated by the breakdown of milk casein using various strains of *Lb. helveticus*, are recognized to be effective angiotensin-converting enzyme (ACE) inhibitors and possess antihypertensive effects (162). The impact of lowering blood pressure may also have repercussions for bone health. Elevated blood pressure has been linked to decreased bone density and loss of bone minerals in women at risk for osteoporosis (163, 164).

Casein phosphopeptides (CPPs) are distinctive peptides present in fermented milk and cheese, which are released through the degradation of proteins by enzymes from different types of bacteria (165). Studies on the hydrolysis of *β*-, αs1-, and αs2-caseins by various strains of *S. thermophilus* have demonstrated that numerous CPPs are released (166). CPPs may have a significant role in bone health by interacting with metal ions such as iron, calcium, and zinc. This interaction can improve the absorption and dissolution of these minerals (167). Consequently, enhanced calcium absorption from CPPs could contribute to better bone health. In research involving animals, CPPs have also been shown to affect the activity of bone-forming cells (152, 168). CPPs can encourage the growth and maturation of osteoblasts (169). The casein-derived peptide valyl-prolyl-proline, produced during fermentation with *Lb. helveticus*, had a beneficial impact on bone turnover in rats (170). Lee et al. (171) indicated that milk products fermented with *Lactobacillus plantarum* A41 and *Lactobacillus fermentum* SRK414 had anti-osteoporotic benefits on post-menopausal rats by regulating markers associated with bone metabolism. Furthermore, it has been hypothesized that milk-derived peptides with antioxidative or anti-inflammatory activities might affect the signaling pathways engaged in bone remodeling (59).

*Kefir* is a fermented dairy beverage that includes BAPs generated during its fermentation (172). The impact of *kefir* peptides (KPs) on bone health has been investigated in multiple studies (Table 2). Chang et al. (173) reported a decrease in pro-inflammatory cytokines present in the serum (IL-1*β*, IL-6, TNF-*α*), and markers of bone resorption (CTX-1, RANKL), while enhanced serum indicators of bone formation (P1NP, OPG, OC), effectively averting bone loss in mice with osteoporosis. Additionally, Lai et al. (31) demonstrated that KPs powder aided in fracture recovery by stimulating the development of bone-forming cells in rats. Similarly, Tu et al. (174) reported an increased bone density in mice. A six-month study involving 40 osteoporotic participants evaluated the effects of *kefir*-fermented milk (1,600 mg) along with calcium bicarbonate (CaCO_3_, 1,500 mg) on bone metabolism. Those who ingested kefir-fermented milk exhibited significant enhancements in BMD as determined by dual-energy X-ray absorptiometry. Patients whose T-scores were above −1 saw a substantial decline in the serum *β* C-terminal telopeptide of type I collagen (β-CTX), and serum OC transitioned from negative to positive post-treatment (66). Nonetheless, the specific effects of KPs remain ambiguous in this study.

Some fermented soybean products include a peptide that might have a beneficial impact on bone health by improving calcium absorption (175) (Table 2). Poly-*γ*-glutamic acid (PGA), a sequence of glutamic acids linked by γ-bonds (176), is present in *B. subtilis*-fermented items, such as *natto* (177) and *kinema* (178). It has been shown that PGA enhances calcium absorption in the intestines of rats. A single administration of PGA resulted in elevated soluble calcium levels in the small intestine (179). These researchers suggested that PGA aids in the creation of a soluble calcium-binding complex, which boosts calcium solubility in the lower small intestine. In humans, Tanimoto et al. (180) examined PGA in a single-blind, randomized, crossover trial, assessing calcium absorption using a double stable isotope technique. They discovered that ingesting two doses of calcium-rich orange juice (200 mg Ca/200 g) and PGA (60 mg/200 g) over a period of 3 to 4 weeks enhanced calcium absorption in healthy post-menopausal women compared to participants who consumed only calcium-fortified orange juice. However, the study had drawbacks, such as a lack of comprehensive details regarding the age, weight, height, and diet of the participants (181). At present, the evidence supporting the function of PGA in enhancing bone health is scarce, and additional research is required to comprehend how it might increase calcium absorption.

Various studies utilizing rodent models have investigated how peptides derived from fermented foods may support bone health. Although peptides were included in these analyses, it is difficult to assert that the impacts on bone metabolism are exclusively attributable to these compounds. For example, Chiang et al. (182) discovered that a 6-week treatment with lactobacilli-fermented soy skim milk aided in diminishing bone loss associated with aging in 13-month-old mice. They proposed that the beneficial effects may arise from both the peptides and other elements such as isoflavones and polysaccharides. BAPs found in fermented dairy products can assist in enhancing bone health by increasing the expression of genes linked to cell growth and the formation of bone cells known as osteoblasts through a specific cellular signaling pathway, which elevates the levels of vital markers for osteoblast formation, including runt-related transcription factor 2 (RUNX2), osteocalcin (OCN), alkaline phosphatase (ALP), and collagen type I alpha 1 chain (COL1A1) (93). Another investigation examined the influence of water-soluble extracts from a long-fermented soybean product from Korea known as *doenjang* on bone metabolism (183). These extracts comprise small proteins generated from the breakdown of larger proteins during fermentation by microorganisms. The water-soluble extracts from *doenjang* facilitated the differentiation of osteoblasts by altering gene expression. Moreover, they enhanced the mineralization of osteoblasts in comparison to the control group. The decrease in tartrate-resistant acid phosphatase activity among cells treated with these extracts suggested that the process of bone resorption by osteoclasts was inhibited. Further studies are required to pinpoint the specific water-soluble peptides that contribute to these effects. It is important to recognize that the biological activity of peptides can be influenced by numerous factors, including the kind of protein, the enzymes utilized, the preparation of the proteins, the proportion of substrate to enzyme, the duration, temperature, pH level, the extent of protein breakdown, the peptide structures, the kinds of amino acids, their molecular weights, and among others (161).

Many of these bioactive compounds whether vitamins, peptides, or polyphenols, interact with the host not only directly but also indirectly through the gut microbiota. This emerging axis, linking dietary components to bone metabolism via microbial modulation, serves as a unifying mechanism that integrates the effects of fermented foods across multiple domains of bone health.

## Gut microbiota for bone health

The human gut microbiota is a diverse community of a high number of microorganisms including bacteria, viruses, fungi, and archaea, residing primarily in the gastrointestinal tract. These microbes play essential roles in metabolism (aiding digestion, extracting nutrients, synthesizing vitamins) such as K and B group, and producing SCFAs; barrier function (maintaining the structural integrity of the gut lining and protecting against pathogen colonization); immune regulation (modulating immune responses, promoting immune tolerance, and influencing systemic inflammation); and neurological effects (affecting the gut-brain axis, impacting cognition, behavior, and neurological health) (72, 184, 185) (Table 2). A balanced gut microbiota is fundamental for overall health, while disturbances, termed dysbiosis, are linked to metabolic, immune, neurodegenerative, and gastrointestinal diseases (72, 184). Emerging research has shown a strong connection between gut microbiota and bone health. This connection has been explained through several mechanisms which have been highlighted in this section. Additionally, interventions altering the gut microbiome such as through diet, prebiotics, or probiotics may positively influence bone density and strength, although findings are still preliminary and sometimes inconsistent (186).

The gut microbiota can enhance bone health through various mechanisms, such as immune responses, hormone synthesis, producing beneficial compounds like SCFAs, aiding in the absorption of nutrients like calcium, and communicating with the brain through neurotransmitter production (187). The microorganisms present in fermented foods can function as probiotics, including *Lactobacillus* and *Bifidobacterium*, generate beneficial peptides and compounds, and convert phenolic compounds into more active forms that may support bone health (188). Fermented foods contain specific microbial groups, especially LAB, that engage with the gut microbiome and can positively influence bone metabolism following consumption (40, 66, 174, 189, 190). Changes in the types or numbers of microorganisms present in the gut can result in dysbiosis, which leads to inflammation in the intestines and modifies gut health (191). When inflammation occurs in the intestines, Th17 cells become activated, resulting in the release of pro-inflammatory substances such as TNF-*α*, IL-1β, IL-6, and IL-17. Marahleh et al. (192) indicated that these pro-inflammatory substances elevate the levels of receptor activator of NF-κB (RANK) ligand (RANKL). RANKL is crucial for the activation of osteoclasts and contributes to bone loss (193, 194). Out of these substances, interleukin 1β is among the most potent pro-inflammatory agents, greatly enhancing bone loss in both laboratory and living models by elevating RANKL levels (195). The activation of TLR5 increases the ratio of RANKL to osteoprotegerin (OPG), which is a natural inhibitor of RANKL, in cells responsible for bone formation, resulting in the production of more osteoclasts and accelerated bone loss (196). RANKL promotes osteoclast maturation by binding to RANK on the surface of osteoclast precursors and stimulates their conversion into osteoclasts that destroy bone (192, 196). It has been demonstrated that diets rich in fermented foods have been demonstrated to enhance bone health by influencing bone formation, owing to the gut bacteria capable of surviving in the digestive tract and interacting with other gut bacteria, which may contribute to improved bone health (197). Despite substantial evidence linking bone health to gut bacteria, many facets of osteoimmunology remain to be thoroughly investigated.

Additionally, gut bacteria might be related to bone health via the endocrine system (198). The initial substance identified that connects the gut and bones was the insulin-like growth factor 1 (IGF-1), primarily produced in the liver in response to food intake and influenced by gut microbiota and specific microbes, such as *Lb. plantarum*, found in fermented foods (197, 199). The IGF-1 assists in bone growth and remodeling (200). There is a possibility that bacteria influence bone health through SCFAs, which are generated when bacteria metabolize fiber, can also result in increased IGF-1 (201). In addition to IGF-1, gut bacteria have been indicated to influence other hormones associated with bone health, including serotonin, parathyroid hormone, glucagon-like peptide 1, and leptin (202). Hydrogen sulfide emerges as another significant factor in microbial endocrinology that regulates bone metabolism (203). Specific intestinal bacteria, such as *Escherichia, Fusobacterium, Desulfovibrio, Streptococcus, Klebsiella, Clostridium, Salmonella*, and *Enterobacter*, convert cysteine into hydrogen sulfide, pyruvate, and ammonia (196, 204, 205). Hydrogen sulfide, a new metabolite generated by gut bacteria, originates from cysteine, homocysteine, and sulfate-reducing bacteria (206), may involve in bone marrow mesenchymal stem cells (207). When there is too little hydrogen sulfide, it disrupts stem cell differentiation, causing irregular calcium flow within the cells and disrupting signaling pathways that are crucial for bone formation (207). Sodium hydrosulfide, a standard source of hydrogen sulfide, was found to reduce RANKL/OPG mRNA levels in human mesenchymal stem cells, which play a role in supporting osteoclasts (208).

Despite the abovementioned findings, it is crucial to note that a recent systematic review has shown that targeting the gut microbiota did not show consistent improvements in bone health. Some studies report positive changes in bone density or strength, while others find no significant effect (186). You et al. have studied relationship between age-dependent microbial change and age-related bone loss in mice and reported that the bone loss associated with age was not dependent on the gut microbiome (209). This directly challenges prior models suggesting microbiota alterations underlie age-associated skeletal deterioration. These findings indicate that there is a need to conduct large longitudinal human cohorts with multi omics, standardized methods, identification of causal microbial strains/metabolites, and assessment of long-term safety and bidirectional signaling.

## Conclusion and future research prospects

Bone health is a multifactorial condition influenced by a combination of dietary components, gut microbiota, and host metabolic processes. This review examined how various bioactive compounds found in fermented foods such as vitamins (K and B groups), polyphenols, bioactive peptides, and fermentation-modified phytates may contribute to bone metabolism through multiple mechanisms (Table 2). These include enhancement of calcium and mineral bioavailability, modulation of oxidative stress and inflammation, and regulation of bone cell signalling pathways involved in osteoblast and osteoclast activity. Additionally, the interplay between fermented foods and gut microbiota particularly the production of SCFAs and immunomodulatory metabolites suggests a potentially important role of the gut-bone axis in skeletal homeostasis.

Despite these findings, the evidence base for fermented foods as modulators of bone health remains less developed than that for traditional approaches such as calcium and vitamin D supplementation or pharmacological interventions. While some preclinical and population studies report associations between fermented food intake and improved bone mineral density or reduced fracture risk, the bioavailability and efficacy of such food-derived compounds are not yet well quantified in humans (40, 210, 211). Moreover, the dose response relationship and long-term impact of fermented food consumption on clinically relevant bone outcomes remain unclear.

A critical gap in the current literature is the limited number of well-designed human clinical trials that evaluate fermented foods in comparison to, or in combination with, conventional bone health interventions. The variability in microbial strains, food matrices, and fermentation processes further complicates the interpretation of findings and the development of standardized dietary recommendations (212–214). Furthermore, while the mechanistic roles of isolated nutrients such as vitamin K2 or polyphenols have been described, few studies investigate the synergistic or antagonistic effects that may occur within complex fermented food systems.

Recent developments in microbiome research and nutritional metabolomics highlight the need for a more personalized approach. Emerging data suggest that individual responses to fermented foods may vary depending on host genetics, baseline nutrient status, and gut microbial composition (215). Additionally, microbial-derived postbiotics such as SCFAs, indoles, or peptidoglycans have been shown to influence bone remodelling pathways, offering a novel direction for future investigation (216). The potential application of synbiotic formulations (fermented foods combined with prebiotics or probiotics) also warrants further study, particularly in populations at high risk for osteoporosis, such as postmenopausal women (217, 218).

To advance this field, future research should focus on establishing the bioavailability and efficacy of fermented food components compared to conventional treatments, identifying strain or product-specific effects on bone health, and evaluating the long-term impact of fermented food consumption on bone quality and fracture outcomes through large scale, controlled human studies. Moreover, interdisciplinary efforts are needed to integrate insights from nutrition science, microbiology, endocrinology, and clinical research to fully elucidate the mechanisms by which fermented foods contribute to skeletal health. Future research should prioritize several critical areas to advance the application of fermented foods in bone health management. First, large-scale randomized controlled trials (RCTs) are essential to confirm the effects of specific fermented foods or bioactives, such as vitamin K2 (MK-7), casein-derived peptides, or fermented soy isoflavones on BMD, turnover markers, and fracture outcomes in humans. Second, stratified analyses based on age, sex, menopausal status, gut microbiota composition, and baseline nutrient levels are needed to identify subpopulations that may benefit most from targeted interventions. Third, comparative studies are warranted to evaluate the synergistic effects of multiple bioactives within complex food matrices versus isolated compounds. Additionally, future investigations should integrate multi-omics approaches (e.g., metagenomics, metabolomics, transcriptomics) to decipher the role of the gut microbiota in mediating host responses to fermented food intake (18).

Technological advances in fermentation also open opportunities for developing functional foods with optimized microbial strains to deliver higher concentrations of bone-beneficial metabolites. Regulatory challenges around standardization and health claims, especially in functional or fortified fermented products, should be addressed to facilitate clinical translation and policy guidance. Furthermore, traditional fermented food systems from regions like India, Japan, Korea, and Africa represent a rich but underexplored source of bioactive compounds, deserving deeper study and possible integration into global dietary strategies (43, 219). Ultimately, bridging preclinical and clinical research through collaborative, interdisciplinary, and longitudinal studies will be essential to unlock the full therapeutic potential of fermented foods in bone health maintenance and osteoporosis prevention.

In conclusion, while fermented foods represent a promising and culturally diverse dietary strategy for supporting bone health, their clinical utility remains to be clearly established. This review underscores the need for targeted research to validate their effectiveness, explore their interaction with the gut microbiome, and position them within the broader context of dietary and therapeutic approaches to osteoporosis prevention and management.

The evidence gathered in this review suggests that incorporating fermented foods into the diet may be a beneficial strategy to complement bone health through natural food-based approaches. However, these effects should be viewed as supportive and not as substitutes for clinically established bone health interventions, such as calcium and vitamin D supplementation or pharmacological treatments when indicated.
